# Edema‐induced changes in tumor cell surviving fraction and tumor control probability in ${\;}^{131}\text{Cs}$ permanent prostate brachytherapy implant patients

**DOI:** 10.1120/jacmp.v14i1.3862

**Published:** 2013-01-07

**Authors:** Than S. Kehwar, Heather A. Jones, M. Saiful Huq, Ryan P. Smith

**Affiliations:** ^1^ Department of Radiation Oncology University of Pittsburgh Cancer Institute, UPMC Cancer Centers Pittsburgh PA USA

**Keywords:** prostatic edema, ${\;}^{131}\text{Cs}$ prostate seed implant, linear quadratic model, surviving fraction, tumor control probability

## Abstract

The study is designed to investigate the effect of edema on the delivered dose, tumor cell surviving fraction (SF), and tumor control probability (TCP) in the patients of prostate cancer who underwent ${\;}^{131}\text{Cs}$ permanent seed implantation. The dose reduction, the SF, and the TCP for edematous prostate implants were calculated for 31 patients who underwent real‐time ${\;}^{131}\text{Cs}$ permanent seed implantation for edema half‐lives (EHL), ranging from 4 days to 34 days and for edema magnitudes ($M_{0}$) varying from 5% to 60% of the actual prostate volume. A dose reduction in ${\;}^{131}\text{Cs}$ implants varied from 1.1% (for EHL=4 days and $M_{0}=5\%$) to 32.3% (for EHL=34 days and $M_{0}=60\%$). These are higher than the dose reduction in ${\;}^{125}I$ implants, which vary from 0.3% (for EHL=4 days and $M_{0}=5\%$) to 17.5% (for EHL=34 days and $M_{0}=60\%$). As EHL increased from 4 days to 34 days and edema magnitude increased from 5% to 60%, the natural logarithmic value of SF increased by 4.57 and the TCP decreased by 0.80. Edema induced increase in the SF and decrease in the TCP in ${\;}^{131}\text{Cs}$ seed implants, is significantly more pronounced in a combination of higher edema magnitude and larger edema half‐lives than for less edema magnitude and lower edema half‐lives, as compared for $M_{0}=60\%$ and EHL=34, and $M_{0}=5\%$ and EHL=4 days.

PACS number: 87.53.Jw

## I. INTRODUCTION

Recently, ${\;}^{131}\text{Cs}$ seeds have been implemented in many centers across USA, including our institution.^(^ ^1^ ^–^ ^3^ ^)^ The design of ${\;}^{131}\text{Cs}$ seed is similar to that of ${\;}^{125}I$ and ${\;}^{103}\text{Pd}$, but has a shorter half‐life of 9.7 days, and slightly higher average photon energy than ${\;}^{125}I$ and ${\;}^{103}\text{Pd}$.^(^ ^4^ ^–^ ^6^ ^)^ The shorter half‐life of ${\;}^{131}\text{Cs}$ can induce differences between planned and delivered doses due to prostatic edema, thus potentially impacting values of dosimetric and radiobiological quantities of interest. Since ${\;}^{131}\text{Cs}$ is a relatively new radioactive source used for prostate permanent seed implants, a number of studies have been done to investigate the effect on edema of ${\;}^{131}\text{Cs}$ permanent seed implants.^(^ ^2^ ^,^ ^3^ ^,^ ^7^ ^–^ ^11^ ^)^

In a recent study, we derived prostate volume changes after prostate brachytherapy from its original volume using US and CT images acquired at day 0, day 14, and day 28 after implant, and found that the prostate edema resolves exponentially with postimplant time.^(^ ^2^ ^)^ The exponential decay of prostatic edema induces a significant amount of change in dosimetric and radiobiological quantities in ${\;}^{131}\text{Cs}$ implants due to its short half‐life of 9.7 days.^(^ ^2^ ^,^ ^3^ ^)^

The present study investigates the effect of edema on dose reduction, tumor cell surviving fraction (SF), and tumor control probability (TCP), for ${\;}^{131}\text{Cs}$ seed permanent prostate implants.

## II. MATERIALS AND METHODS

### A. radiobiological model

For protracted irradiation, the cell surviving fraction, S(D), can be written by:

(1) $$S(D)=\text{exp}[-\alpha D(t)-\beta q(t)D{\left(t\right)}^{2}]$$

where α and β represent the single‐track and intertrack cellular radiosensitivities. The q(t) is a dose protraction factor to account for the sublethal damage repair for an implant where dose rate decays exponentially,^(^ ^12^ ^)^ given by:

(2) $$\begin{array}{l} q(t)=\left[2{\left(\mathrm{\lambda t}\right)}^{2}/\left\{{\left(\mathrm{\mu t}\right)}^{2}(1-\lambda^{2}/\mu^{2}){\left(1-e^{-\mathrm{\lambda t}}\right)}^{2}\right\}\right][\left\{e^{-(\lambda +\mu )t}\right\}\\ +\left\{\mathrm{\mu t}(1-e^{-2\mathrm{\lambda t}})/(2\mathrm{\lambda t})\right\}-\left\{(1+e^{-2\mathrm{\lambda t}})/2\right\}] \end{array}$$

where λ and μ are the decay constant of radioactive source and repair constant of sublethal damage, respectively. These can be defined by $\lambda =\ln(2)/t_{1/2}$ and $\mu =\ln(2)/t_{\text{rep}}$ where $t_{1/2}$ and $t_{\text{rep}}$ are the half‐life of radionuclide used in the implant and repair half‐time of sublethal damage, respectively.

In permanent implants, the dose rate at a point ‘P’ by decaying radioactive sources is a simple exponential function of time:

(3) $$R_{p}(t)=R_{P}(0)\;\text{exp}(-\mathrm{\lambda t})$$

and the total dose delivered to point ‘P’ is given by:

(4) $$D_{P}=R_{p}(0)\int_{0}^{\infty }\text{exp}(-\lambda t)\text{dt}$$

where $R_{p}(0)$ is the initial dose rate at point ‘P’ in the tumor.

Antipas et al.^(^ ^13^ ^)^ had shown that in permanent implants, the biologically effective dose rate delivered to the tumor cells falls with time due to radionuclide decay, and extends to the point at which the biological dose rate falls to the critical dose rate, where it is equal to or less than the tumor cell repopulation. The time interval between the implantation time (day 0) and the time at which the dose rate reaches this critical value is called the effective treatment time ($t_{\text{eff}}$).^(^ ^13^ ^)^ The $t_{\text{eff}}$ provides a measure of the time over which tumor cell kill is ensured and is given by:

(5) $$t_{\text{eff}}=-(1/\lambda )\ln\{0.693/({\mathrm{\alpha R}}_{P}(0)T_{p})\}$$

where $T_{p}$ is the tumor cell potential doubling time.

By truncating Eq. (4), the dose delivered at point ‘P’ in time ‘t’ can be given by:

(6) $$D_{p}(t)=R_{p}(0)\int_{0}^{t}\text{exp}(-\mathrm{\lambda t})\text{dt}$$

where *t* varies from 0 to $t_{\text{eff}}$.

Using Eq. (6) into Eq. (1), S(D) at ‘P’ for cumulative dose at P, can be written as:

(7) $$S_{p}(D_{eff})=\text{exp}[-\alpha R_{p}(0)\int_{0}^{teff}\text{exp}(-\mathrm{\lambda t})\text{dt}-\mathrm{\beta q}(t){\left\{R_{p}(0)\int_{0}^{teff}\text{exp}(-\mathrm{\lambda t})\text{dt}\right\}}^{2}]$$

In a permanent prostate implant, due to induced prostatic edema, the prostate volume and source locations become function of time and, thus, instantaneous dose at point ‘P’ will not be a simple exponential function of time, but is given by the following relation,^(^ ^8^ ^)^ which accounts for an edema induced dose reduction at time ‘t’. The dose delivered at point ‘P’ in time ‘t’ is given by:

(8) $$D_{p}(t)=R_{p}(0)\int_{0}^{t}[\text{exp}(-\mathrm{\lambda t})/{\left\{1+M_{0}\text{exp}(-\lambda_{e}t)\right\}}^{\tau /3}]\text{dt}$$

where $M_{0}$ is the initial magnitude of the edema (is defined by $M_{0}=(V_{0}-V_{p})/V_{p}$, where $V_{p}$ and $V_{0}$ are the pre‐implant volume and postimplant volume at day 0, respectively), $\lambda_{e}$ is the edema decay constant (defined by $\lambda_{e}=\ln(2)/t_{e}$, where $t_{e}$ is the edema half‐life (EHL)), and the exponent τ was determined to have a value of 2.20 by Chen et al.^(^ ^14^ ^)^ for the Model CS‐1 ${\;}^{131}\text{Cs}$ sources.

Uniform dose distributions are not achievable in permanent implants; therefore, to deduce the TCP directly from nonuniform dose distributions, Webb and Nahum^(^ ^15^ ^)^ modified the TCP model for nonuniform clonogenic cell density and nonuniform dose distributions, and is given by:

(9) $$\text{TCP}=\prod_{i=1}^{n}\text{exp}[-\rho_{i}V_{i}S_{i}(D_{\text{eff}\_i})]=\text{exp}[-\sum_{i=1}^{n}\rho_{i}V_{i}S_{i}(D_{\text{eff}\_1})]$$

where *n*, $\rho_{i}$, *and* $V_{i}$ are the number of voxels within the prostate volume, the initial clonogenic cell density and voxel volume of ith voxel, respectively. The $S_{i}(D_{\text{eff}\_i})$) is the surviving fraction for average cumulative dose in ith voxel. In the calculations of $S_{i}(D_{\text{eff}\_i})$), it was assumed that the ith voxel received an average dose rate of $R_{i}(0)$ at day 0. With the help of Eq. (8), the $S_{i}(D_{\text{eff}\_i})$) for ith voxel can be written by:

(10) $$\begin{array}{l} S_{i}(D_{eff\_i})=\text{exp}[-\alpha R_{i}(0)\int_{0}^{t}[\text{exp}(-\lambda t)/{\left\{1+M_{0}\text{exp}(-\lambda_{e}t)\right\}}^{\tau /3}]\text{dt}\\ -\beta q(t)\{R_{i}(0)\int_{0}^{t}\left[\text{exp}(-\lambda t)/{\left\{1+M_{0}\text{exp}(-\lambda_{e}t)\right\}}^{\tau /3}]\text{dt}\}{\;}^{2}\right] \end{array}$$

If it is assumed that the clonogenic cell density ρ is uniform throughout the tumor volume, then Eq. (9) may have the form given by:

(11) $$\text{TCP}=\text{exp}[-\sum_{i=1}^{n}\rho V_{i}S_{i}(D_{\text{eff}\_i})]$$

From Eq. (11), S(D) can be written as:

(12) $$S(D)=(1/V)\sum_{i=1}^{n}V_{i}S_{i}(D_{\text{eff}\_i})$$

where *V* is the prostate volume with edema at day 0.

This is the same equation as discussed in earlier articles.^(^ ^16^ ^,^ ^17^ ^)^

Equation (12) can be rewritten in terms of exponentially decaying edema magnitude by

(13) $$S(D)=(1/V)\sum_{i=l}^{n}\left[V_{pi}\{1+M_{0}\exp(-\lambda_{e}t)\}S_{i}(D_{eff\_i})\right]$$

where $V_{pi}$ is the pre‐implant volume of ith voxel, and the TCP for pre‐implant prostate volume can be written as:

(14) $$\text{TCP}=\text{exp}[-\rho \;V_{p}\;S(D)]$$

where $V_{p}$ and ρ are the prostate volume and clonogenic cell density before implant procedure, respectively.

### B. radiobiological parameters

A set of radiobiological parameters is required for the S(D), TCP and $t_{\text{eff}}$ calculations. The values of these parameters were taken from previously published reports^(^ ^2^ ^,^ ^18^ ^)^ and are as follows: $\alpha =0.15\;{\text{Gy}}^{-1}$, $\beta =0.05\;{\text{Gy}}^{-2}$, α/β=3.0 Gy, $T_{p}=42$ days, $\mu =61.6\;d^{-1}$ (i.e., $\mu =\ln(2)/t_{\text{rep}}$, here repair half‐life $\mu =\ln(2)/t_{\text{rep}}$, $\rho =1\beta y\;10^{6}$,^(^ 18 ^)^ and edema decay constant $\lambda_{e}=0.0713\;d^{-1}(i.e.,\;\lambda_{e}=\;\ln(2)/t_{1/2\_\text{edema}},$ where $t_{1/2\_\text{edema}}$ is the half‐life of edema decay with a value of 9.72 days).^(^ ^2^ ^)^

### C. Patients

Thirty‐one patients of prostate cancer, who received a prescribed dose of 115 Gy to the prostate by permanent ${\;}^{131}\text{Cs}$ seed implants, were analyzed in this study. Details of the implant procedure, technique, and seed loading were discussed in previous studies.^(^ ^12^ ^)^ Briefly, for each patient the transrectal ultrasound (US) was used to obtain images of the prostate prior to the implantation, as well as pre‐ and postneedle insertion prior to ${\;}^{131}\text{Cs}$ seed implantation. The positioning of the needles and seeds in the needles were defined with the guidance of US images obtained pre‐ and postneedle insertion. The postimplant CT images were also obtained on the day of the implant (day 0) and at day 14 and day 28. Contouring of the prostate on US and CT images, and seed localization and analysis of the data, was performed by the same individual for each patient. The seed locations were generated for US images of preneedle and preseed (postneedle) prostate volumes, and for the CT images of postimplant prostate volumes at days 0, 14, and 28.

## III. RESULTS & DISCUSSION

For 31 patients,^(^ ^2^ ^)^ the average initial magnitude of edema ($M_{0}$) developed immediately after the implantation on day 0, was found to be 22.76%±5.99% (range from 5.15% to 84.47%) for preneedle US and postneedle US images, and 19.81%±4.94% (range from 5.36% to 63.23%) for preneedle US images and postseed implant CT images at day 0 (p>0.05, Student's t–test).^(^ ^2^ ^)^ The values of EHL, determined by comparing the volumes obtained for preneedle US and postseed implant CT images at days 14 and 28, were found to range from 3.64 days to 34.48 days with a mean of 9.72±8.31 days (mean±1 SD).^(^ ^2^ ^)^ In another study, the EHL was reported to vary from 4 to 30 days.^(^ ^19^ ^)^

To account for the fast decaying ${\;}^{131}\text{Cs}$ seeds, the $t_{\text{eff}}$ was calculated using Eq. (5) with an assumption that the prescribed dose of 115 Gy is delivered uniformly to the prostate, and was found to be 60.36 days, which is very close to the 61 days recommended in AAPM TG 137 Report for ${\;}^{131}\text{Cs}$ prostate implants.^(^ ^18^ ^)^

**Figure 1:**
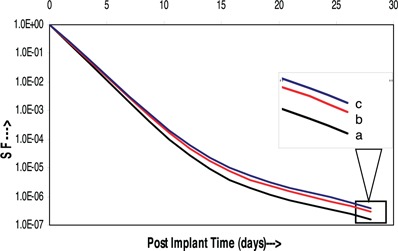
Changes in SF with postimplant time. The line ‘a’ represents SF for prescription dose without edema correction, line ‘b’ represents for calculated SF using Eq. (21) for day 0 CT images, and line ‘c’ for individual CT images obtained at day 0, day 14, and day 28.

Figure 1 shows the relationship between mean surviving fractions and postimplant time, where line ‘a’ represents the SF for the prescribed dose distributed uniformly throughout the prostate volume without considering the effects of edema, line ‘b’ for the SF calculated taking into account the initial CT volumes obtained at day 0 and magnitude of the edema with half life of 9.72 days, and the line ‘c’ represents the SF for the seed locations generated for individual postimplant CT volumes obtained at days 0, 14, and 28. There were statistically significant differences in the SFs at days 14 and 28 for: (i) prescribed uniform dose and that for initial CT volumes (p=0.02 and 0.01, respectively, Z‐test), and (ii) prescribed uniform dose and that for individual postimplant CT volumes (p=0.02 and 0.03, respectively, Z‐test). However, there were no statistically significant differences between the SFs for initial CT volumes and individual postimplant CT volumes (p=0.11 and 0.16, respectively, Student's t‐test); hence, it is clear that Eq. (13) calculates the SF accurately. The plots in Fig. 1 revealed that the SF calculated using prescribed dose without edema resolution correction overestimates the results than that of actual implants.

The TCP values calculated for the dose delivered in time $t_{\text{eff}}$, using Eq. (14) and individual postimplant CT volumes obtained at day 28, have no statistically significant differences (p>0.05), and were found to ranged from 0.55 to 0.99 with a mean and SD of 0.76±0.14.

It was mentioned earlier that EHLs calculated for these patients were found to vary from 3.64 days to 34.48 days.^(^ ^2^ ^)^ Hence, to estimate the effect of different EHL on the SF and the TCP, calculations were performed using Eqs. (13) and (14) for EHLs of 4, 10, 15, 20, 25, and 34 days for all patients.

**Figure 2:**
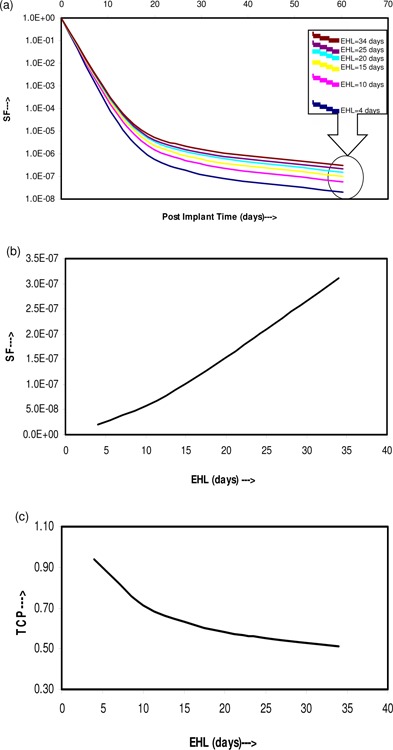
Change in the SF with post implant time: (a) plot of SF versus postimplant time for different EHL; (b) plot of SF calculated at $t_{\text{eff}}$ versus EHL ranging from 4 days to 34 days; and (c) plot of TCP with EHL ranging from 4 days to 34 days corresponding to the SF of (b).

Figure 2(a) shows the plots of the mean values of SF versus postimplant time for edema half‐lives of 4, 10, 15, 20, 25, and 34 days, and shows that the mean value of SF increased steadily with increasing EHL. Figure 2(b) represents a plot of the dependence of SF on EHL, calculated at $t_{\text{eff}}$, which shows that as EHL is increased from 4 days to 34 days, the natural logarithmic value of SF increased by 2.71, The corresponding changes in TCP resulting from the changes in the SF are shown in Fig. 2(c), which shows a decrease in the mean TCP from 0.94 to 0.51 for increasing EHL from 4 days to 34 days.

**Figure 3:**
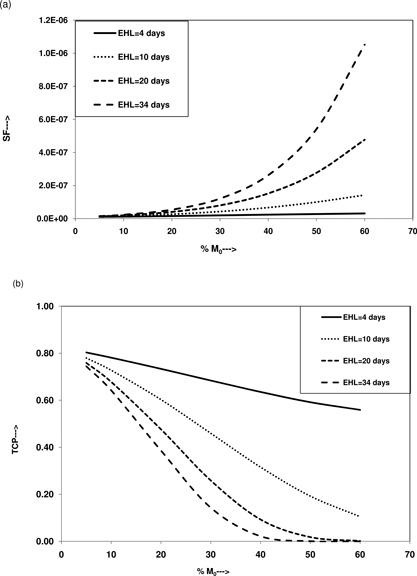
Schematic demonstration of role of EHL on SF and TCP calculated postimplant time at $t_{\text{eff}}$: (a) plots of SF as a function of magnitude of edema at different EHL, and (b) plots of TCP as a function of magnitude of edema at different EHL.

The values of $M_{0}$ for these patients, obtained from preneedle US images and postseed implant CT images at day 0, were found to range from 5.36% to 63.23%. Hence, the calculations of SF and TCP for all 31 patients were done for edema magnitudes of 5%, 10%, 20%, 40%, and 60%. The plots of SF versus $M_{0}$ and TCP versus $M_{0}$ are shown in Figs. 3(a) and 3(b) for EHL ranging from 4 days to 34 days. Figures 3(a) and 3(b) illustrate that the SF increases and the TCP decreases steadily with increasing values of EHL and $M_{0}$. At EHLs of 4 days, 10 days, 20 days, and 34 days, as $M_{0}$ increases from 5% to 60%, the natural logarithmic value of SF increases by 1.04, 2.44, 3.54, and 4.26, respectively, and the TCP decreases by 0.24, 0.67, 0.76, and 0.74, respectively. Similarly, at $M_{0}$ of 5%, 10%, 20%, 40%, and 60%, as EHL increased from 4 days to 34 days, the natural logarithmic value of SF increased from −18.34 to −18.02, −18.21 to −17.59, −17.97 to −16.74, −17.56 to −15.15, and −17.29 to −13.77, respectively, and the TCP decreased from 0.80 to 0.74, 0.78 to 0.64, 0.73 to 0.39, 0.64 to 0.02, and 0.56 to 0.00, respectively. The change in the SF and TCP is more dramatic when the values of EHL and $M_{0}$ are greater than 10 days and 30%, respectively, and is more pronounced for extreme values of EHL and $M_{0}$, such as 34 days and 60%, respectively.

The dose reductions in ${\;}^{131}\text{Cs}$ and ${\;}^{125}I$ implants as a function of EHL and $M_{0}$ are listed in Table 1. It is seen in Table 1 that the window of dose reduction increases with increasing $M_{0}$, and found to be more pronounced in ${\;}^{131}\text{Cs}$ permanent implants compared to the ${\;}^{125}I$ implants, and becomes worst for the extreme combination of EHL of 34 days and $M_{0}$ of 60%. In this study, the edema‐induced dose reduction was computed with postimplant time and found that for an average edema magnitude of 20%, as the EHL is increased from 4 days to 34 days, the dose reduction increased from 4.27% to 11.27% in ${\;}^{131}\text{Cs}$, and 1.03% to 5.98% in ${\;}^{125}I$ implants.

**Table 1:** Percentage dose reduction for different edema magnitude with edema half‐lifes.

	$M_{o}$									
*EHL*	*5%*		*10%*		*20%*		*40%*		*60%*	
*(days)*	${\;}^{131}\mathrm{Cs}$	${\;}^{125}I$	${\;}^{131}\mathrm{Cs}$	${\;}^{125}I$	${\;}^{131}\mathrm{Cs}$	${\;}^{125}I$	${\;}^{131}\mathrm{Cs}$	${\;}^{125}I$	${\;}^{131}\mathrm{Cs}$	${\;}^{125}I$
4	1.08%	0.26%	2.15%	0.52%	4.27%	1.03%	8.41%	2.04%	12.41%	3.03%
10	1.88%	0.59%	3.74%	1.19%	7.41%	2.36%	14.55%	4.67%	21.42%	6.93%
20	2.48%	1.04%	4.95%	2.08%	9.79%	4.13%	19.18%	8.17%	28.17%	12.10%
34	2.86%	1.51%	5.70%	3.01%	11.27%	5.98%	22.03%	11.79%	32.29%	17.45%

## IV. CONCLUSIONS

Results of the present study show that the short half‐life of ${\;}^{131}\text{Cs}$ seeds causes drastic edema‐induced dose reduction in the implants, because approximately 80% of the prescribed dose is delivered during first three weeks of the implant. As edema magnitude becomes larger and decays more slowly, the dose reduction is more pronounced and, consequently, more tumor cells survive the treatment of ${\;}^{131}\text{Cs}$ seed implants. Edema induced increase in the SF and decrease in the TCP in ${\;}^{131}\text{Cs}$ seed implants is significantly more pronounced in a combination of higher edema magnitude and larger edema half‐lives than for less edema magnitude and lower edema half‐lives, as compared for $M_{0}=60\%$ and EHL=34, and $M_{0}=5\%$ and EHL=4 days.
